# Troubles psychotiques précipités par le mariage : étude de trois observations

**DOI:** 10.11604/pamj.2013.14.146.2660

**Published:** 2013-04-13

**Authors:** Masmoudi Jaweher, Mohamed Faouzi Kammoun, Feki Inès, Baati Imen, Sallami Rim, Jaoua Abdelaziz

**Affiliations:** 1 Service de Psychiatrie A, CHU Hédi Chaker 3029, Sfax, Tunisie

**Keywords:** Mariage, psychose, nuptiale, marriage, psychosis, Bridal

## Abstract

Le mariage est un évènement très investi dans notre culture arabo-musulmane. Il présente une situation à grande charge émotionnelle et ayant un vécu stressant. C'est ainsi qu'il peut être à l'origine de la décompensation de certains troubles psychiatriques. Ce moment particulier de déclenchement de la pathologie peut altérer significativement l'adaptation familiale et sociale du patient en question, le rendant dépendant en partie ou en totalité à une institution. Dans ce travail, nous proposons d’étudier certains facteurs psychiques, sociaux et culturels pouvant aboutir à la précipitation des manifestations psychotiques par le mariage. Il s'agit de l’étude de trois observations cliniques, deux hommes et une femme, hospitalisés dans le service de psychiatrie A du CHU Hédi Chaker de Sfax et qui ont développés des manifestations psychotiques de façon concomitante à leur mariage. La durée moyenne de survenue des crises a été de vingt ans, le diagnostic retenu a été celui de trouble bipolaire dans deux cas et d'une schizophrénie indifférenciée chez le troisième patient. L’évolution s'est faite vers une chronicisation de deux malades et une dépendance institutionnelle dans le troisième cas. La précipitation des troubles psychotiques par le mariage, reste un phénomène en relation intime avec les composantes culturelles, elles-mêmes sont déterminantes dans la prise en charge ultérieure de ces patients.

## Introduction

Le mariage est un terme qui désigne généralement l'union légale d'une femme et d'un homme. C'est une relation qui a plusieurs implications : psychologique, socio familiale, religieuse, etc… Le mariage réunit plusieurs intérêts: le respect de la nature de l’être humain dans le sens d'assouvir ses désirs, et de préserver l'espèce humaine, dans un cadre bien codifié; un intérêt social, du fait du respect des normes de la société et ses apparences; un intérêt religieux, dans la mesure où cette union est considérée comme sacrée par la majorité des religions. Il existe, bien entendu, des contraintes à cette relation, tels que: le choix du conjoint, qui dépend de plusieurs facteurs; le vécu psychologique des nouveaux mariés, et le symbole du mariage pour chacun; les exigences financières et les responsabilités requises pour le nouveau statut social. Le mariage devrait être conforme aux modèles offerts par la société en vigueur. Ce qui n'est pas toujours évident. En plus, il devrait pouvoir être consommé. Tous ces facteurs, transforment le mariage en un moment de fragilité particulière l'assimilant à un facteur de stress d'importance capitale. La période nuptiale, peut se trouver ainsi à l'origine de la décompensation de certains troubles psychotiques. La fréquence des réactions psychotiques aiguës est liée selon certains auteurs [1–3] à des facteurs socioculturels. Dans ce travail, et à travers l'illustration de trois observations cliniques, nous proposons d’étudier: les facteurs psychiques, sociaux et culturels pouvant transformer le mariage en un moment propice pour la précipitation des manifestations psychotiques et l'influence du déclenchement de la pathologie, à ce moment particulier, sur la prise en charge ultérieure.

## Patients et observations

### Cas clinique 1

Monsieur A est âgé de 70 ans, originaire et demeurant à Sfax. Il est issu d'un mariage non consanguin et est le huitième d'une fratrie de neuf (5 garçons et 4 filles). Il fait partie d'une famille modeste et assez conservatrice. Son père a été maçon. Sa mère a été femme au foyer. Il a été scolarisé jusqu'au baccalauréat, et à l’âge de 20 ans, il a réussi un concours pour recrutement d'administrateur dans un établissement public. Depuis son jeune âge, Monsieur A avait de l'affection pour une amie de classe, une voisine Mademoiselle Z, issue d'une famille nettement plus aisée que la sienne. A l’âge de vingt-deux ans, Monsieur A fidèle à ses sentiments, et à sa bien-aimée, a demandé Mademoiselle Z eu mariage, demande qui n'a pas été bien accueillie par la famille de mademoiselle Z et surtout par sa grand -mère. Cependant, et sous la pression de la jeune fille, la famille a fini par céder et accepter cette union, au prix d'une dot nettement plus élevée (le triple) par rapport aux conventions de l’époque. Monsieur A a subvenu très difficilement aux exigences de sa belle-famille, et la date du mariage a été fixée. Un mois avant la date présumée, la grand-mère de Mademoiselle Z, celle qui a tant lutté contre cette union décéda. La famille de l’épouse est en deuil, et plusieurs membres de la famille ont proposé de reporter le mariage. Mais, Monsieur A s'est opposé, et la fête a été célébrée avec peu de manifestations joviales. Pendant la soirée, un désaccord concernant certains détails d'organisations s'est produit entre les deux familles. La nuit des noces Monsieur A a présenté une euphorie, une puissance inhabituelle et un désir non maîtrisable. Il a eu plusieurs rapports sexuels avec sa nouvelle mariée, ce qui lui a causé une déchirure vulvaire ayant nécessité une assistance chirurgicale urgente. Monsieur A de son côté, a été hospitalisé d'urgence en psychiatrie. Il a été irritable, anxieux avec une agitation psychomotrice et un délire riche. Les thèmes du délire ont été variables (puissance, grandeur et ensorcellement): il disait qu'on l'a ensorcelé, que ses confrères lui veulent du mal, et que le soleil est enceint de lui. L'amélioration du tableau clinique, sous neuroleptiques, a été passagère et aléatoire. Ce qui a motivé le recours à plusieurs séances de sismothérapie au bout desquelles une amélioration clinique a été observée, avec une sortie de l'hôpital et une rupture ultérieure des neuroleptiques. Monsieur A a eu six enfants (3 Garçons et 3 filles) et il a repris ses activités professionnelles.

Dix-sept ans après le premier épisode et à l'occasion des fiançailles de sa fille aînée avec un cousin, il a présenté à nouveau une décompensation maniaque. Depuis, les accès se sont répétés pour devenir de plus en plus proches. Le diagnostic de trouble bipolaire a été retenu et le patient a été mis sous neuroleptiques et thymorégulateurs. Durant son suivi le patient a présenté une vingtaine d'accès maniaques, rendant le patient, pour des années, dépendant ou presque des structures institutionnelles psychiatriques.

A l’âge de 65 ans, Monsieur A a présenté pour la première fois un accès mélancolique avec caractéristiques catatoniques. La prise en charge fut difficile et laborieuse d'autant plus qu'il a développé, quelques années avant son accès mélancolique de troubles de la conduction cardiaque. L’évolution ultérieure a été marquée par une détérioration cognitive importante limitant son autonomie et le rendant totalement dépendant de son épouse.

### Cas clinique 2

Madame H, âgée de 44 ans, d'un milieu rural, a été scolarisée jusqu'en troisième année primaire. Elle est l'aînée d'une fratrie de sept (6 filles et un garçon). Son père est décédé à la suite d'une hémorragie digestive. Sa mère a été une vieille dame présentant des séquelles d'une hémiplégie consécutive à un accident vasculaire cérébral. Madame H est issue d'une famille pauvre. Elle n'avait comme ressources financières qu'une allocation familiale trimestrielle. Elle a quitté l’école très tôt pour travailler dans une usine de confection de tapis, participant ainsi à subvenir aux besoins matériels de sa famille. A l’âge de vingt ans, son père a décidé de la marier à un agriculteur aisé, mariage longuement contesté par Madame H. Un mois avant son mariage et suite à un conflit avec son père concernant le mariage, elle a présenté un état d'agitation psychomotrice avec une hyperactivité, une insomnie et des fugues nocturnes, nécessitant le recours à l'usage des neuroleptiques, traitement arrêté au bout de quelques jours. La fête du mariage s'est déroulée sans incidents, ainsi que la nuit des noces. Le troisième jour de son mariage, elle a été hospitalisée en psychiatrie pour état d'agitation, insomnie, déambulation nocturne, hyperactivité et un délire. Les thèmes de délire ont été essentiellement de persécution et de préjudice. Vu la grande agitation, Madame H a bénéficié de cinq séances de sismothérapie, puis elle a été mise sous neuroleptiques, traitement arrêté aussitôt sortie de l'institution. Le diagnostic retenu a été une psychose aigue nuptiale. Cinq mois plus tard, et suite à un conflit avec sa belle-famille, elle a tenté de mettre le feu à la maison. Ce qui a motivé son hospitalisation d'office.

Deux ans plus tard, de façon concomitante à une menace maritale de divorce, elle a effectué une tentative de suicide, altruiste par égorgement, en associant sa petite fille âgée de huit mois, seule Madame H a pu être sauvée. Elle a été condamnée à cinq ans de prison. Durant ce séjour en prison elle a été hospitalisée à deux reprises en psychiatrie, pour état d'agitation psychomotrice et hétéro-agressivité. De plus, son mari a obtenu le divorce. A l’âge de vingt-six ans, Madame H s'est remariée à un monsieur nettement plus âgé qu'elle et travaillant comme marin pêcheur. Un garçon naîtra de cette union. Ce second mariage de Madame H n'a pas duré puisque son nouveau mari est décédé au bout de quelques années.

Madame H est revenue alors vivre avec sa famille, mais le vécu de meurtrière qui l'a marqué auprès de son entourage a aggravé leur conflits, et a abouti à son rejet de la part de ses parents. Ainsi, au bout de quelques mois de vie commune avec sa famille, Madame H a commis une deuxième tentative de suicide altruiste, en se jetant dans un puits avec son enfant; seule la patiente a pu être sauvée. Elle a été admise de nouveau en psychiatrie, dans un état d'inhibition psycho motrice totale. Actuellement elle est à sa 15ème année d'hospitalisation, bien stabilisée sous sel de lithium, et manifeste une profonde réticence chaque fois qu'on lui propose l'idée de sortir. Sa famille, à son tour, ne manifeste aucun désir d'accompagner sa sortie.

### Cas Clinique 3

Monsieur B est âgé de 55 ans et issu d'un mariage consanguin de premier degré. Son père a été décrit comme strict et conservateur; soit Imam d'une mosquée, et ayant élevé ses enfants selon les traditions et les Lois de la Religion Islamique. C'est le troisième d'une fratrie de cinq (trois garçons et deux filles). Il a été diplômé de l’école coranique Mosquée Zitouna et a travaillé comme instituteur. Monsieur B a été toujours décrit comme ayant un caractère timide, introverti, et un manque de confiance en lui. Ses relations avec l'autre sexe ont été réduites uniquement au cadre professionnel. A l’âge de trente ans, Monsieur B a fait la connaissance d'une collègue, Mademoiselle A, une jeune fille issue d'une famille bien renommée de la région. Il a demandé Mademoiselle A au mariage, demande rapidement acceptée.

La fête du mariage s'est déroulée selon les traditions. Cependant, la fête a dû être interrompue, suite à une dispute importante entre deux invités et l'annonce de l'hospitalisation inopinée du frère aîné de monsieur B, en chirurgie. La même nuit, nuit des noces, la découverte de la perte prémaritale de la virginité de mademoiselle A a profondément bouleversé Monsieur B. Ceci ne l'a pas empêché de consommer le mariage. Au cours de la septième journée post nuptiale, monsieur B a “entendu” une profonde voix intérieure, lui disant: “tu dois te venger de ta femme, elle t'a trahit, elle t'a trompé, venges toi d'elle”. Puis les hallucinations se sont enrichies; il a dit avoir vu, et entendu le président de la République lui ordonnant de quitter sa femme ou de la torturer. Il a dit avoir vu sa femme avec une tête de loup, de lion ou de serpent. Monsieur B est devenu de plus en plus, halluciné et délirant avec des thèmes de persécution, de préjudice et d'ensorcellement, et avec un automatisme mental. Il a été mis sous neuroleptiques classiques sédatifs et incisifs. Le mariage de Monsieur B n'a duré qu'une année, le fruit de ce mariage a été un garçon. Ce dernier a été élevé par sa mère, qui s'est remariée.

En 2003, soit après dix-huit années du déclenchement de sa maladie, et à l'occasion d'un jour de fête religieuse, monsieur B sortant faire un tour, a rencontré un petit garçon, le fils des voisins et s'est approché de lui. Le garçon effrayé, l'a traité de fou en disant “éloigne-toi, espèce de fou”; Monsieur B s'est acharné sur le petit en le frappant par une grosse pierre au niveau de la tête occasionnant sa mort, fait toujours dénié par le malade. Depuis cette date, monsieur B a été hospitalisé en psychiatrie. Aucun membre de sa famille même son fils âgé actuellement de vingt ans, n'a éprouvé le désir de lui rendre visite ni de le prendre en charge. L’évolution a été marquée par des moments d'atténuation et des moments d'exacerbation des symptômes délirants. Le diagnostic de schizophrénie paranoïde a été retenu devant la réunion de critères requis (délire, dissociation, autisme’).

## Discussion

Le mariage, phénomène social universel, est vécu dans une atmosphère de gaieté et de catharsis, mais derrière cette joie, il y a tout un vécu stressant. Le degré de stress attribué au mariage selon l’échelle de Holmes et Rahé est de 50 par rapport à un maximum de 100 [4]. Le ravivement des pulsions peut tourner au tragique avec l’éclosion de manifestations psychotiques dites nuptiales et le drame peut faire place au plaisir convoité et minutieusement préparé. Il existe, bien entendu, une variabilité inter personnelle, en ce qui concerne la réaction face à un facteur de stress. Ainsi, l'importance accordée au mariage dépend, non seulement de la personnalité sous-jacente de l'individu, mais aussi de son histoire personnelle, et des événements vécus. Dans ce contexte, les troubles psychotiques précipités par le mariage sont des troubles qui peuvent être assimilés à un état psychotique aigu avec facteur de stress marqué: le mariage. La relation entre la pathologie psychotique et le mariage est basée sur un critère temporel; un délai d'au moins un mois entre le mariage et le déclenchement des troubles est nécessaire. Cependant, les auteurs soulignent l'impossibilité d'affirmer que le lien soit forcement causal [5].

En Tunisie, le profil des patients à risque de développer une psychose nuptiale, est un sujet jeune, de sexe masculin, issu d'un milieu rural, de niveau scolaire primaire, de bas niveau socio-économique et indemne d'antécédents psychiatriques [4, 6]. D'autre part, les facteurs socio culturels ont un intérêt capital dans la précipitation des troubles psychotiques par le mariage. En effet, Langen a remarqué que “la psychose de la lune de miel” est plus fréquente chez les touristes de nationalité Japonaise que chez les autres nationalités. Ceci serait selon lui, expliqué par les particularités socio culturelles du mariage au Japon [7]. De même, dans son étude sur les troubles psychotiques précipités par le mariage dans une communauté ultra-orthodoxe, Fisch a conclu à l'importance des facteurs éducatifs et culturels dans le déclenchement de ces troubles [8].

En ce qui concerne nos patients: monsieur A, a vu ses troubles se précipiter par le mariage. Plusieurs facteurs ont participé à le rendre plus vulnérable: le type d’éducation reçue (milieu conservateur); son niveau socio-économique, nettement plus inférieur que celui de mademoiselle Z; le refus de la famille de mademoiselle Z pour cette union; les contraintes matérielles imposées par la famille de mademoiselle Z; le décès de la grand-mère de mademoiselle Z avec les menaces de reporter le mariage; le désaccord entre les deux familles concernant les détails de l'organisation de la fête du mariage. Monsieur A devrait se défendre contre son sentiment d'impuissance devant sa belle-famille rejetant, sentiment assimilable à une atteinte narcissique; une atteinte à sa virilité, au sens large.

Les relations matrimoniales, depuis les sociétés archaïques déjà, avaient de profondes implications économiques: la pratique de la dot, avec toutes ses implications psychiques et économiques, s′est largement perpétuée jusqu′il y a peu de temps, et demeure encore d′application dans certains pays. La dot de mademoiselle Z a été très élevée, compliquant la tache davantage, pour monsieur A. Le mariage constitue, dans la majorité des cas de notre société, la première occasion d'un contact sexuel entre les deux partenaires. Le jeune mari doit faire preuve de sa virilité. Cette situation est très angoissante, et explique la fragilité particulière des jeunes époux [9]. Monsieur A cherchant peut être, à confirmer sa virilité, à plusieurs niveaux, s'est déchaîné sur le plan sexuel. Ce déchaînement a été manifesté dans le cadre d'une décompensation maniaque. Plus tard, c’était à l'occasion de la célébration des fiançailles de sa fille que monsieur A a présenté une nouvelle décompensation maniaque, soit après un intervalle libre de bonne qualité de dix-sept ans. Il s'agirait d'une réactivation de sa propre problématique, voire d'une reviviscence d'une problématique œdipienne. Le mariage de Monsieur A a duré, contrairement aux autres patients. Il a un support socio familial de bonne qualité. Madame H n'a pas pu surmonter l’épreuve difficile du mariage. Plusieurs facteurs ont pu participer à la rendre vulnérable au développement des manifestations psychotiques: le milieu familial peu contenant, un père âgé et une mère malade; le faible niveau socio-économique; le faible niveau d’éducation, son non-consentement pour le mariage. Un mariage non désiré, sera ainsi transformé en une épreuve difficilement surmontable, et redoutée. C'est ainsi, que madame H aurait épuisé toutes ses capacités de défenses, et s'est précipitée dans la psychose. Les conflits conjugaux n'ont fait qu'aggraver son sentiment d’être atteinte dans son intégrité, avec un dégoût conséquent pour la vie. Le suicide altruiste exprimerait, son désir de sauver ses enfants du sort que leur sera réservé. Le vécu de meurtrière qui accompagne l'image de Madame H a voué à l’échec toute tentative d'insertion sociale ou familiale.

Monsieur B, a reçu une éducation très stricte. La découverte de la défloration pré maritale de mademoiselle A, aurait été le principal facteur qui a précipité le déclenchement des troubles psychotiques. L'analyse de l'observation de Monsieur B met en évidence l'importance des facteurs socio culturels dans la précipitation des troubles psychotiques par le mariage: du point de vue religieux, L′Islam considère la sexualité que dans le cadre du mariage. Dans ce cadre, la sexualité est un acte de foi. Toutes les formes de la sexualité en dehors du mariage, sont condamnées sévèrement, tant pour l'homme que pour la femme. Au sein des péchés dits “majeurs”, la sexualité en dehors du cadre marital apparaît parmi les plus graves [10]; du point de vue social, seule la femme musulmane connaîtra le déshonneur des aventures préconjugales. Dans notre contexte socio culturel, l'exigence de virginité de la femme au moment du mariage, est une garantie “d'honneur” pour les familles. Et de la “pureté” de l’épouse pour le mari. Haffani et al. ont souligné, à travers 10 observations de femmes hospitalisées en psychiatrie, le rôle de la perte de la virginité dans le déclenchement des troubles psychopathologiques aigus en Tunisie. Ces troubles étaient essentiellement représentés par des psychoses délirantes aiguës avec un syndrome délirant de persécution et d'influence à mécanisme hallucinatoire auditif [11]. L’épouse de monsieur B a été loin d’être l’épouse idéale, et surtout du fait fa sa perte prémaritale de sa virginité. Elle l'aurait profondément déçue dans ses attentes. Monsieur B déstabilisé, ne savait plus quoi faire: dénoncer Mademoiselle A au prix d'un scandale familial ou accepter le “péché” contre tous les principes qu'il a reçus depuis son enfance. Le résultat de ces stress accumulés a été la bascule dans la psychose. Le geste meurtrier qu'a commis monsieur B peut être assimilé, à l'assassinat du fils. Le désir de monsieur B serait d’être libéré de ce fils qui représente la ficelle qui ne cesse de le relier à cette femme “pécheresse” et de compenser une atteinte narcissique profonde.

## Conclusion

Le mariage, phénomène en relation intime avec les composantes socioculturelles et religieuses de la société ainsi qu'avec le vécu du sujet, semble être déterminant dans l’éclosion des troubles psychiatriques, particulièrement psychotiques, et à évolution parfois chronique. Ce qui peut constituer un tournant dramatique dans la vie de sujets “victimes”. Pour prévenir ces décompensations, citons par exemple; une bonne préparation des jeunes concernant les différents aspects du mariage, grâce à un programme d’éducation planifiée à l’école; faire participer les mass-médias pour passer des messages sensibilisateurs voire éducateurs; créer une consultation prénuptiale, dédramatisant, parfois démystifiant le mariage, et parfois permettant de détecter des signes de fragilité, de détresse, voire de décompensation psychiatrique.
